# Effect of Loading Rate and Initial Strain on Seismic Performance of an Innovative Self-Centering SMA Brace

**DOI:** 10.3390/ma15031234

**Published:** 2022-02-07

**Authors:** Yigang Jia, Bo Zhang, Sizhi Zeng, Fenghua Tang, Shujun Hu, Wenping Chen

**Affiliations:** 1 School of Civil Engineering and Architecture, Nanchang University, Nanchang 330031, China; jiayigang999@sina.com (Y.J.); zhangbo_tujian@nerin.com (B.Z.); tfh2450748@163.com (F.T.); hushujun@ncu.edu.cn (S.H.); 2 Design and Research Institute, Nanchang University, Nanchang 330031, China; 3 Zhongmei Engineering Group Ltd., Nanchang 330001, China; 4 Jiangxi Huaye Special Engineering Technology Co., Ltd., Nanchang 330001, China; huayejiagu2022@163.com

**Keywords:** shape memory alloy (SMA), self-centering SMA brace, loading rate, initial strain, energy dissipation coefficient

## Abstract

In order to improve the energy dissipation capacity and to reduce the residual deformation of civil structures simultaneously, this paper puts forwards an innovative self-centering shape memory alloy (SMA) brace that is based on the design concepts of SMA’s superelasticity and low friction slip. Seven self-centering SMA brace specimens were tested under cyclic loading, and the hysteresis curves, bond curves, secant stiffness, energy dissipation coefficient, equivalent damping coefficient, and the self-centering capacity ratio of these specimens were investigated, allowing us to provide an evaluation of the effects of the loading rate and initial strain on the seismic performance. The test results show that the self-centering SMA braces have an excellent energy dissipation capacity, bearing capacity, and self-centering capacity, while the steel plates remain elastic, and the SMA in the specimens that are always under tension are able to return to the initial state. The hysteresis curves of all of the specimens are idealized as a flag shape with low residual deformation, and the self-centering capacity ratio reached 89.38%. In addition, both the loading rate and the initial strain were shown to have a great influence on the seismic performance of the self-centering SMA brace. The improved numerical models combined with the Graesser model and Bouc–Wen model in MATLAB were used to simulate the seismic performance of the proposed braces with different loading rates and initial strains, and the numerical results are consistent with the test results under the same conditions, meaning that they can accurately predict the seismic performance of the self-centering SMA brace proposed here.

In major earthquakes, buckling-restrained brace frames [1] and eccentrically braced frames [2] demonstrate high stiffness, high ductility, and good energy dissipating capacity. However, conventional steel frames may develop severe residual deformations and structural damage when subjected to strong earthquakes [3,4] In recent years, the concept of a re-centering mechanism for civil structures has been proposed; it was considered to be an efficient way to reduce residual deformation and to further improve the energy dissipating capacity of these structures [5,6]. Therefore, many kinds of steel frames with self-centering devices were proposed, and have demonstrated the advantages of high stiffness, low residual deformation, and easy construction [7,8].

Shape memory alloy (SMA) wire is a new type of smart material with a shape memory effect and superelasticity effect that can return to its initial shape after experiencing a strain value of 0.06 with negligible residual deformation upon unloading [9,10]. A great deal of research shows that self-centering dampers equipped with SMAs have emerged as energy-dissipating and re-centering candidates for civil structures [11,12,13,14,15,16]. For example, Xue et al. [11] proposed a self-centering friction damper with SMA wires and friction devices, and this damper had an excellent energy dissipation capacity. Qiu and Zhu [12] investigated the behavior of novel self-centering SMA braces equipped with SMA as a key component. Xu et al. [13] illustrated an innovative self-centering link beam with steel rods and SMA rods to provide the re-centering force. Fang et al. [12,13] presented a novel type of self-centering steel connection with SMA rings, which showed satisfactory energy dissipation and excellent self-centering capability. Hu et al. [16] indicated that a new self-centering brace had the advantages of good seismic performance, a high self-centering capacity, and zero damage. To examine the influence of self-centering SMA dampers on the seismic performance of civil structures, Li et al. [17] studied the seismic performance of a six-story steel frame with an innovative re-centering damper, which had an outstanding re-centering capacity. Fan et al. [18] further confirmed that the prepressed spring self-centering braces in the steel frame could mitigate post-earthquake residual deformation. 

However, some studies revealed that the seismic performance of the self-centering braces mentioned above could be affected by the mechanical properties of the SMA wires themselves. Zhou et al. [19] conducted fatigue testing on SMA wires that were 0.5 mm and 1.0 mm in diameter and indicated that the initial strain and loading frequency had a great effect on the mechanical properties of the SMA wires. Yan et al. [20] revealed the influence of cyclic numbers, the loading rate, and the strain amplitude on SMA wires. Qian et al. [21] carried out the cyclic loading of SMA wires by changing the variable amplitudes and loading rates. Hu et al. [22] investigated the effect of the cyclic number, strain amplitude, initial strain, and loading rate on an SMA wire with a 1 mm diameter, and the results of the mechanical property evaluation indicated that the cyclic number was less clear but that the initial strain and loading rate should be emphasized. Therefore, the loading rate and initial strain are two of the main factors that could be used to determine the seismic performance of a self-centering brace with SMA wires.

This paper presents experimental research on the effect of the loading rate and the initial strain of a self-centering SMA brace under cyclic loading, and the hysteresis curves, bond curve, secant stiffness, energy dissipation coefficient, equivalent damping coefficient, and self-centering capacity ratio (ratio between super-elastic displacement and maximum displacement) of the braces are analyzed in detail. Then, the modified mechanical model of the self-centering SMA brace is developed based on the improved Grasser model program and the Bouc–Wen model, and the MATLAB/SIMULINK toolbox is used to conduct the simulation, allowing the accuracy of the numerical results to be compared to the test results.

## 1. Basic Properties of Self-Centering SMA Brace

**Figure 1:**
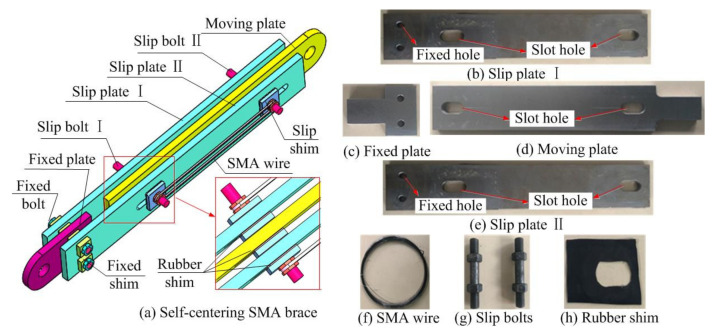
Schematic diagram of self-centering SMA brace.

As shown Figure 1, self-centering SMA braces mainly include three parts: a slip component, a fixed component, and SMA wires. The slip component is composed of a moving plate, slip plate I and slip plate II, which are connected by slip bolt I and slip bolt II. The fixed component is constituted by the moving plate, the fixed plate, and slip plate II, which has a fixed bolt connection. The SMA wires are set on both ends of the two slip bolts to provide the energy dissipating capacity and elastic restoring force, which provides the special property of superelasticity. Moreover, rubber shims are placed on both sides of the slip shim to reduce the friction coefficient, and the slip bolt passes through the moving plate, slip shim, slip plateI, slip shim, and slip plate II in sequence. It is suggested that the fixed shims be installed between the moving plate and fixed plate and slip plate II. In addition, two slot holes are located at both ends of the moving plate, and there are two slot holes in the same position on slip plate II and on the fixed plate.

**Figure 2:**
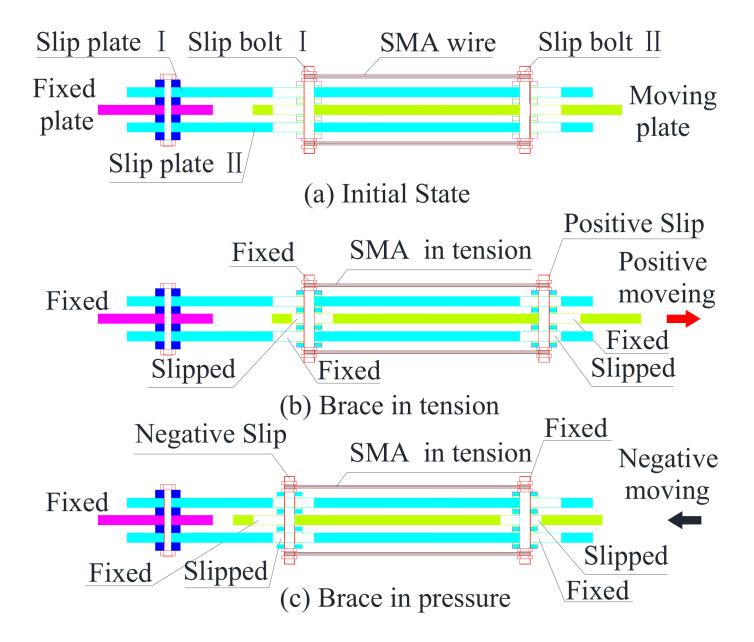
(**a**) initial state, (**b**) brace in tension, (**c**) brace in pressure, Work principle of self-centering SMA brace.

Figure 2 illustrates the work principle of a self-centering SMA brace. As shown Figure 2b, when the fixed plate is fixed and the brace is in tension conditions at the moving plate, slip bolt II is fixed in the moving plate and slipped in the slip plates, and slip bolt I is fixed in the slip plates and slipped into the moving plate simultaneously. As shown Figure 2c, when the brace is in pressure conditions at the moving plate, the slip bolt I is fixed in the moving plate and slipped in the slip plates, slip bolt II is fixed in the slip plates and slipped into the moving plate simultaneously. During the positive and negative movements, the SMA wires are always subjected to elongation, thus increasing the ductility, energy dissipating capacity, and self-centering capacity. In ideal conditions, when the external load is unloaded, the SMA wires can almost force the slip component return back to the initial state, and only slight residual deformation in observed in the self-centering SMA brace, which is mainly caused by the very low friction coefficient of the rubber shims.

## 2. Test Investigation

### 2.1. Test Specimens

**Figure 3:**
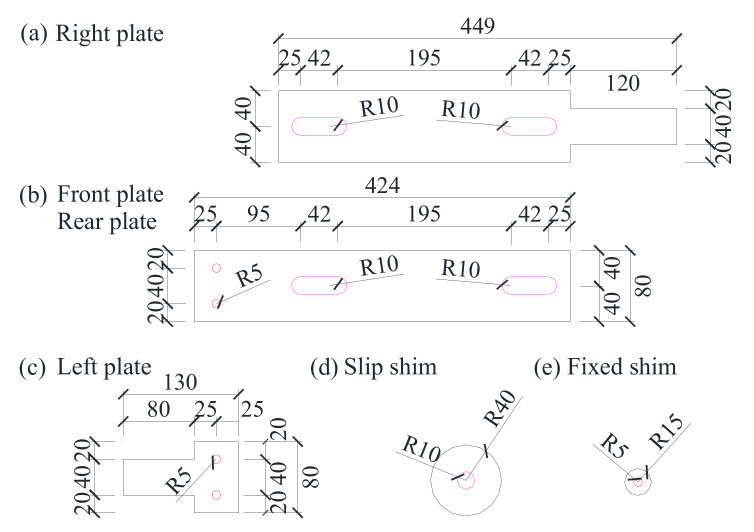
(**a**) right plate (**b**) front plate/rear plate (**c**) left plate (**d**) slip shim (**e**) fixed shim. Details of the self-centering SMA brace.

In total, the comparison of seven self-centering braces with different loading rates and initial strains are tested in this paper. All of the braces are composed of a slip component, fixed component, and SMA wires, as shown in Figure 3. Slip plateI has a cross-section that is 449 mm × 80 mm in size and a thickness of 15 mm. The moving plate and slip plate II have a cross-section that is 424 mm × 80 mm in size and a thickness of 8 mm. The fixed plate has a cross-section that is 130 mm × 80 mm in size and a thickness of 15 mm. The radii, *R*, of the fixed hole, slip shim, and fixed shim are 5 mm, 15 mm, and 40 mm, respectively. The thicknesses, *t_f_*, of the slip shim, fixed shim, and rubber shim are 5 mm, 3 mm, and 1 mm, respectively. The radius of the slot hole is 10 mm and has a length of 42 mm.

The parameters of the specimens are listed in Table 1. The specimens consist of four parameters: the torque value of the slip bolt, the SMA area, the loading rate of the SMA wire, and the initial strain of the SMA wire. The torque value and SMA area of the specimens are 10 N·M and 43.96 mm^2^. For example, the specimen “SCB-12-25” represents the brace at a loading rate of 0.0012 s^−1^ and an initial strain amplitude of 0.25%. The effect of the loading rate of the self-centering SMA brace can be revealed by specimens SCB-12-0, SCB-18-0, SCB-24-0, and SCB-36-0, while the influence of the initial strain is reflected by specimens SCB-12-0, SCB-12-25, SCB-12-50, and SCB-12-100.

### 2.2. Material Properties

#### 2.2.1. SMA Wire

**Figure 4:**
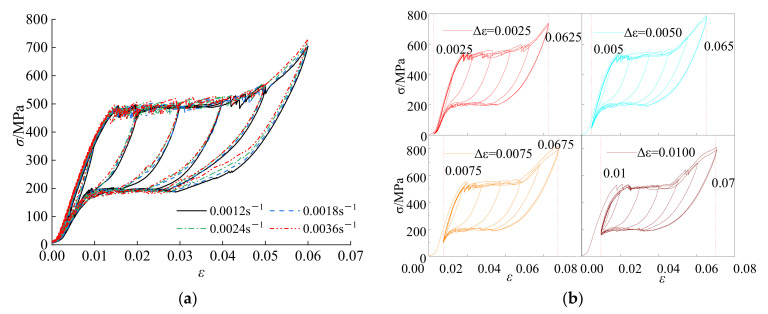
(**a**) Loading rate; (**b**) initial strain. Hysteresis curves of SMA wires with different influencing factors [22].

The tested SMA wire with a diameter of 1.0 mm diameter was obtained from Gao’an SMA Material Co., Ltd. According to data offered by the manufacturer, the chemical composition of the SMA wire in terms of weight was close to Ni-55.96%, Ti-43.9835%, H-0.0005%, Cr-0.0070%, Co-0.003%, C-0.005%, Fe-0.006%, Cu-0.006%, and others-0.029%. Tests on the mechanical properties of the SMA wire at different loading rates and initial strains under cyclic loading were carried out, and the hysteresis curves are shown in Figure 4 [22].

**Figure 5:**
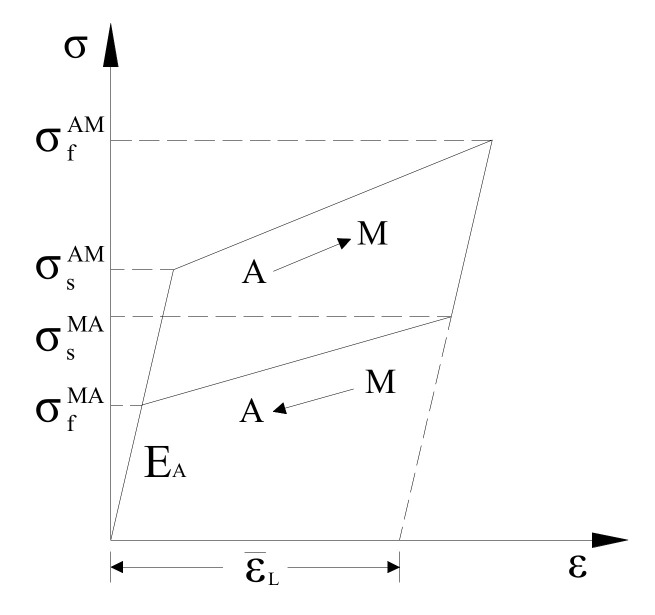
Constitutive model of SMA wire [22].

The mechanical properties in the constitutive model of the SMA wire comprise six parameters, i.e., $\sigma_{s}^{AM}$, $\sigma_{f}^{AM}$, $\sigma_{s}^{MA}$, $\sigma_{f}^{MA}$, $\bar{\varepsilon_{L}}$, and $E_{A}$ [10], as shown in Figure 5. Based on the mechanical properties obtained from Figure 4, the main parameters of SMA wires with different influencing factors are shown in Table 2.

#### 2.2.2. Steel Plate

The tested steel plates with thicknesses of 8 mm and 15 mm were all made of Q345B steel and had a nominal design yield strength of 345MPa. The samples that were obtained from the steel plates were subjected to standard metallic tensile tests, and the mechanical properties that were measured are listed in Table 3.

### 2.3. Test Setup

**Figure 6:**
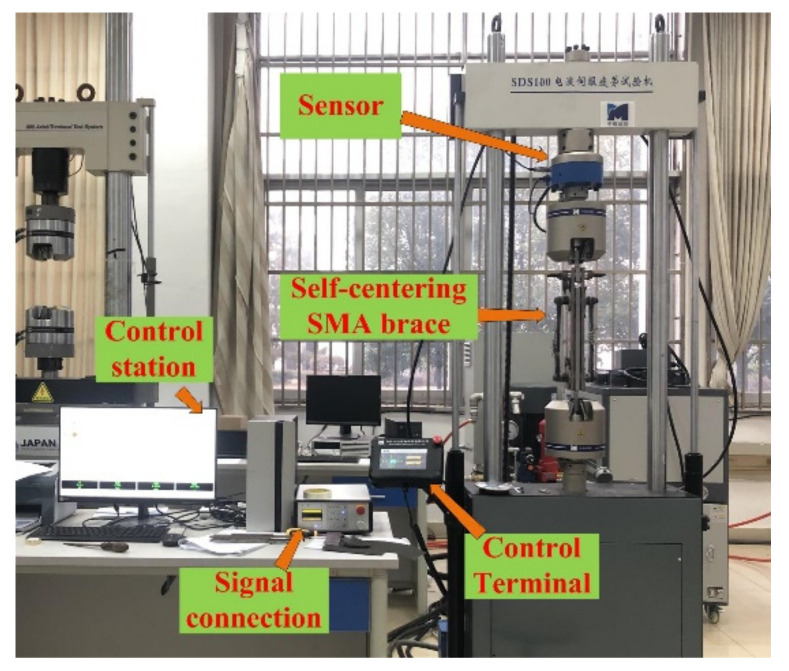
Diagram of the experimental setup.

The cyclic loading test was carried out using an SDS100 fatigue test machine at the Engineering Mechanics Experiment Center, Nanchang University. The test setup was composed of four parts: the sensor, control station, control terminal, and signal connection, as shown in Figure 6. The maximum hydraulically driven load that the SDS100 can apply is 100 kN, which is applied with a precision of 0.01 kN. The lower fixture and upper fixture were connected to the fixed plate and slip plateI of the self-centering SMA brace, respectively. The load and displacement of the test specimens were directly recorded by the sensor. All tests were conducted at 27 °C.

### 2.4. Test Cases

The tested cases of self-centering SMA brace specimens are shown in Table 4. The cases one through four studied the effect of the different loading rates, and the loading rates were set as 0.0012 s^−1^, 0.0018 s^−1^, 0.0024 s^−1^, and 0.0036 s^−1^, respectively, whereas the initial strain was zero, and the loading cycle was one. The first case as well as cases five to seven considered the effect of the initial strains, which were 0.0025, 0.0050, 0.0075, and 0.0100, and all tests were loaded for at a loading rate of 0.0012 s^−1^ for one cycle. In addition, a total of seven loading displacement ranges were set for all of the specimens: 1.20 mm, 2.40 mm, 4.80 mm, 7.20 mm, 9.60 mm, 12.00 mm, and 14.40 mm, which were set successively [22], and the corresponding strain amplitudes were 0.005, 0.01, 0.02, 0.03, 0.04, 0.05, and 0.06.

## 3. Test Results

### 3.1. Hysteresis Curves

**Figure 7:**
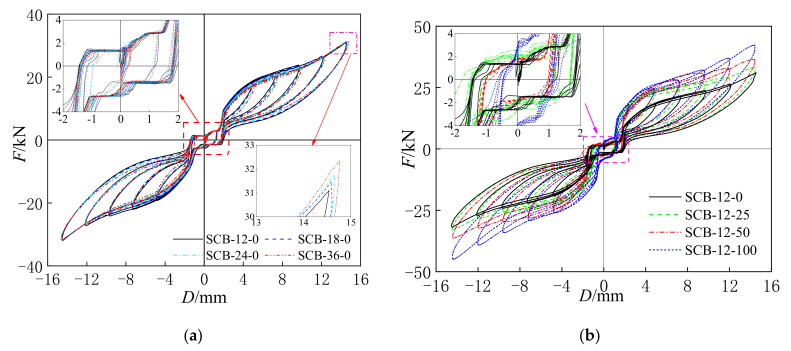
(**a**) Loading rate; (**b**) initial strain. Hysteresis curves of self-centering SMA brace specimens.

Figure 7 shows the hysteresis curves of the self-centering SMA brace specimens with different loading rates and initial strains. The hysteresis curves mainly consist of three successive phases: the initial slip phase, the rapidly increasing stress–strain phase, and the rapidly decreasing phase. The figure clearly shows that all of the hysteresis curves exhibit a high self-centering capacity and ideal flag-shape hysteresis with low residual deformation and slip strength. The self-centering capacity and ideal flag-shape hysteresis are primarily caused by the SMA wires, while low residual deformation and slip strength are induced by the slip component. In addition, the rectangular loops around the origin point are caused by low residual deformation and slip strength.

(1) Effect of loading rates: Figure 7a plots the hysteresis curves of the SCBs with different loading rates. It can be seen that the maximum axial force *F* and residual deformation *D*_1_ are 31.08 kN, 31.49 kN, 31.75 kN, 32.23 kN and 1.97 mm, 1.90 mm, 1.87 mm, 1.85 mm at the loading rates of 0.0012 s^−1^, 0.0018 s^−1^, 0.0024 s^−1^, and 0.0036 s^−1^, respectively. The ultimate axial force increased gradually, and the residual deformation decreased slightly as the loading rate increased.
(2) Effect of initial strains: The hysteresis curves of the SCBs with different initial strains are shown in Figure 7b. The maximum axial forces *F* of the SCBs were 31.08 kN, 33.21 kN, 36.80 kN, and 42.24 kN at the loading rates of 0.25%, 0.50%, 0.75%, and 1.00%, respectively, which significantly increased as the initial strain increased. In addition, the results also indicate that the residual deformation *D*_1_ at the loading rates of 0.25%, 0.50%, 0.75%, and 1.00% were 1.97 mm, 1.89 mm, 1.69 mm, and 1.53 mm, respectively, which was mainly caused by the increase in the initial stress and ultimate stress of the SMA wires [22].

### 3.2. Bond Curves

**Figure 8:**
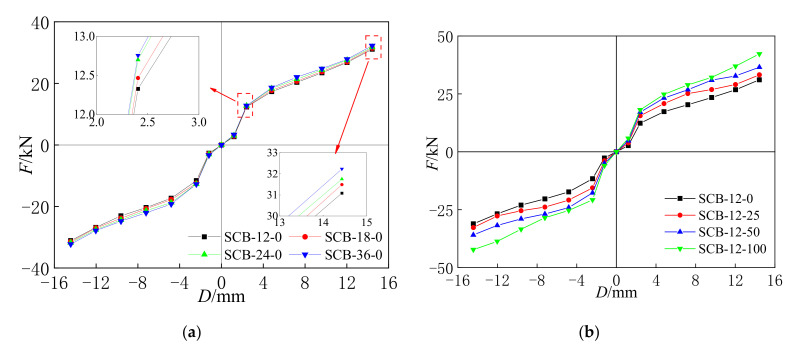
(**a**) Loading rate; (**b**) initial strain. Bond curves of self-centering SMA brace specimens.

The bond curves in Figure 8 were obtained from the results in Figure 7. The bond curves in the initial slip phase have a smaller initial stiffness, and the bearing capacity and initial stiffness gradually increased in the increasing phase. Finally, the bearing capacity of the specimens increased slowly and reached the maximum axial force, and the slope of the bond curve began to decrease in the decreasing phase. All of the bond curves are origin-symmetric under axial tension and axial compression. In addition, as shown in Figure 8a,b, the bearing capacity of specimens SCB-12-0, SCB-18-0, SCB-24-0, SCB-36-0 increased as the loading rate increased, and the same conclusion can be obtained for the initial strain rates for specimens SCB-12-0, SCB-12-25, SCB-12-50, and SCB-12-100.

### 3.3. Secant Stiffness

In this paper, the stiffness degradation of the self-centering SMA brace is presented by the secant stiffness coefficient, *K_si_*, which can be calculated by:

(1) $$K_{si}=\frac{\left|F_{i,\max}\right|+\left|-F_{i,\min}\right|}{\left|D_{i,\max}\right|+\left|-D_{i,\min}\right|}$$

where *F_i_*,max, *F_i_*,min, *D_i_*,max, and *D_i_*,min represent the maximum axial force, minimum axial compression, maximum displacement, and minimum displacement at the *i*-th hysteretic cycle under the load displacement of *i*.

**Figure 9:**
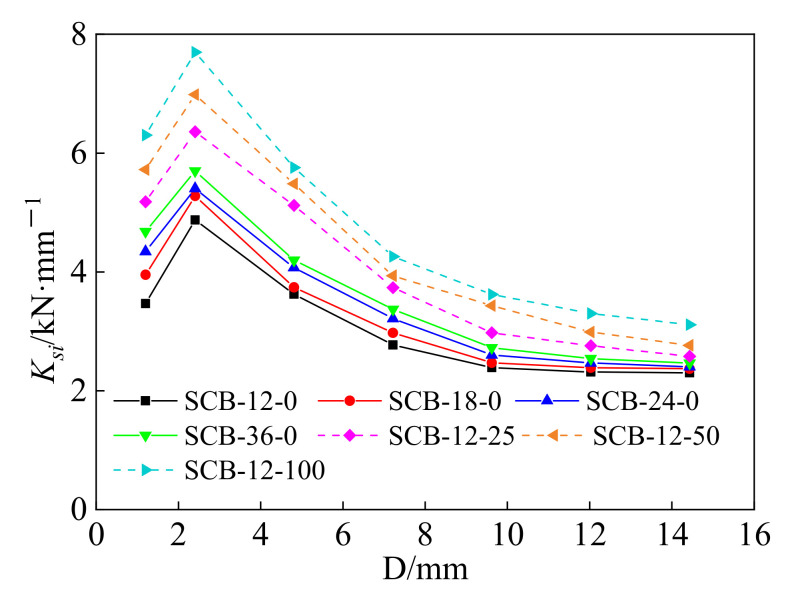
Secant stiffness of self-centering SMA brace specimens.

Figure 9 shows the secant stiffness curves of all of the test specimens. The secant stiffness coefficient decreases after reaching the maximum value as the applied displacement increases and the reduction rate gradually decreases. The initial increase in the secant stiffness coefficient is mainly because of the slip friction of the slip components. In addition, as the loading rate and initial strain increase, it can be seen that the secant stiffness coefficients of the self-centering SMA braces increase gradually.

### 3.4. Energy Dissipation Coefficient

**Figure 10:**
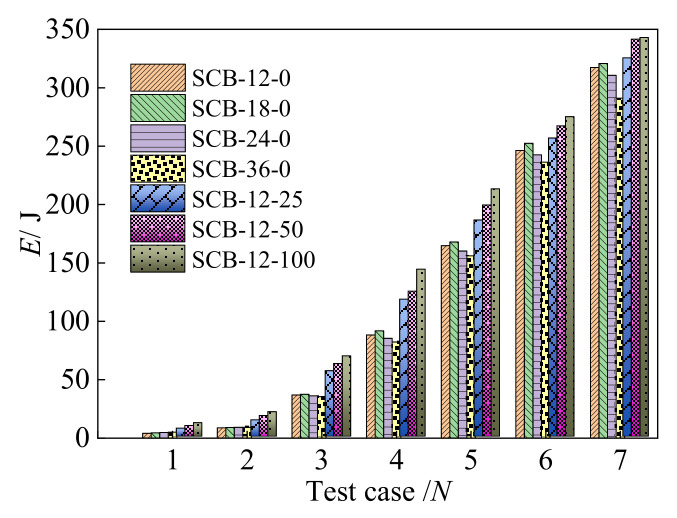
Energy dissipation coefficient of specimens.

The different energy dissipation coefficients, *E_i_*, which are calculated by the enclosed area of the hysteresis curve for each specimen, are shown in Figure 10. The maximum *E_i_* of the specimens SCB-12-0, SCB-18-0, SCB-24-0, and SCB-36-0 are 317.37 J, 320.80 J, 310.73 J, and 291.13 J, respectively. An increase in the *E_i_* in each test case can be observed when the loading rates are increased from 0.0012 s^−1^ to 0.0018 s^−1^, an a gradual decrease occurs as the loading rate increases to 0.0036 s^−1^, which is influenced by the pinch phenomenon in the hysteresis curves [22]. In addition, an increase in the *E_i_* for specimens SCB-12-0, SCB-12-25, SCB-12-50, and SCB-12-100 can be clearly seen under different test cases where there is an increase of initial strain from 0 to 0.01, which was mainly caused by the increase in the hysteresis areas. Therefore, the energy dissipation capacity can be effectively increased by increasing the initial strain, but the influence law of the loading rate for the self-centering SMA braces is uncertain, meaning that further research should be on the equivalent damping coefficient.

### 3.5. Equivalent Damping Coefficient

**Figure 11:**
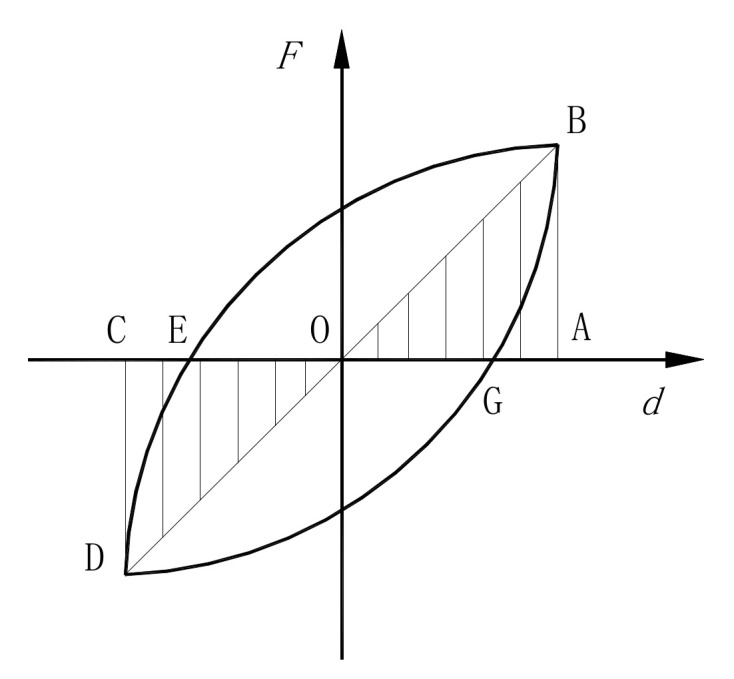
Calculation of the energy dissipation coefficient.

The equivalent damping coefficient, *ξ_eq_*, is also an important parameter that can be used to evaluate the energy dissipation capacity, which can be calculated as follows [23]:

(2) $$\xi_{eq}=\frac{1}{2\pi }\frac{S_{EBG}+S_{DEG}}{S_{OBA}+S_{ODC}}$$

where *S_EBG_* and *S_DEG_* represent the areas of the closed geometrical figures EBG and DEG, which are enclosed by the vertical axial force and horizontal displacement of the coordinates, and *S_OBA_* and *S_ODC_* represent the areas of the triangles OBA and ODC in Figure 11.

**Figure 12:**
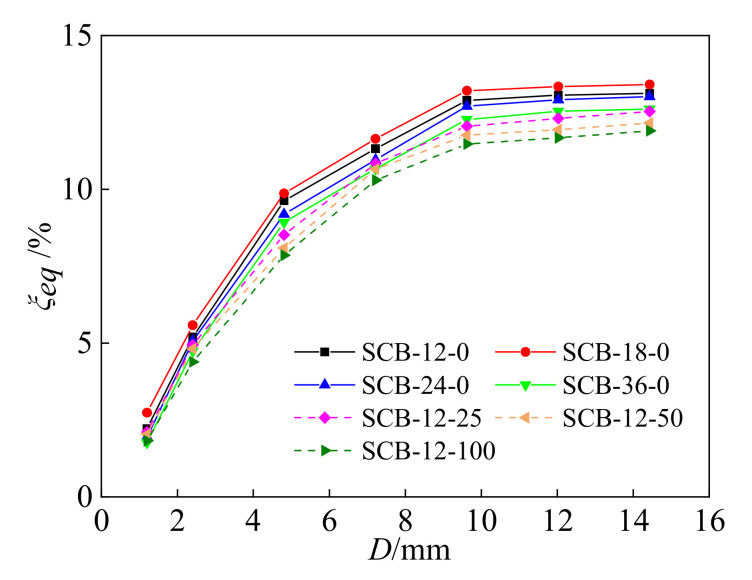
Equivalent damping coefficient of self-centering SMA brace specimens.

For all of the test specimens, the equivalent damping coefficient during the loading-unloading process is presented in Figure 12. The equivalent damping coefficient of all of the specimens increases as the applied displacement increases. For the specimens with different loading rates, the equivalent damping coefficient of specimen SCB-18-0 is higher than that of specimens SCB-12-0, SCB-24-0, and SCB-36-0; this is mainly caused by the hysteresis area and energy dissipation value. Compared to specimen SCB-12-0, SCB-12-25, SCB-12-50, and SCB-12-100 have a larger maximum axial force *F* and nearly the same energy dissipation value *E_i_*, resulting in a lower equivalent damping coefficient as the initial strain increases.

### 3.6. Self-Centering Capacity Ratio

**Figure 13:**
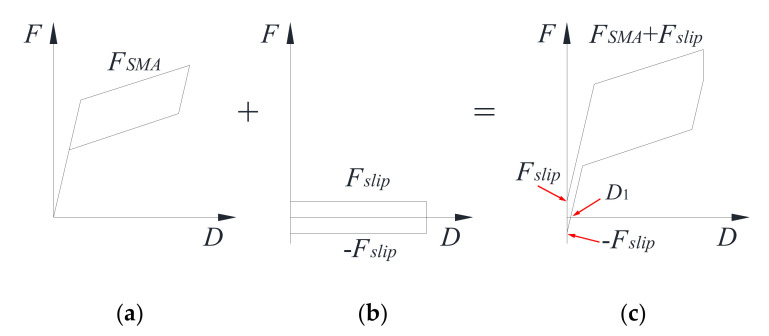
(**a**) SMA, (**b**) slip component, and (**c**) self-centering SMA brace force–displacement curve of self-centering SMA brace.

The proposed self-centering SMA brace is mainly composed of a slip component and re-centering component, and consequently, the axial force, *F_SCB_*, can be written by:

(3) $$F_{\mathrm{SCB}}=F_{\mathrm{SMA}}+F_{slip}$$

where *F_SMA_* and *F_slip_* are the forces of the SMA wires and slip, as shown in Figure 13.

The self-centering capacity ratio, *δ*, is an important parameter that can be used to evaluate the re-centering capacity of self-centering SMA braces and can be calculated as follows:

(4) $$\delta =\frac{D-D_{1}}{D}$$

where *D* is the maximum applied displacement, and *D*_1_ is the residual displacement.

Based on the test results in Section 3.1, the maximum *F_u,SCB_*, *F_slip_*, *F_SMA_*, *D*, *D*_1_, and *δ* of each specimen can be concluded and are shown in Table 5. The *F_slip_* was obtained by the vertical force of the rectangular loops around the origin point in Figure 7, and the *F_SMA_* is equal to the *F_u,SCB_* minus the *F_slip_*. By increasing the loading rate, the *F_u,SCB_* and *δ* increased, while the *F_slip_* and *D*_1_ decreased. In addition, a larger initial strain also resulted in a greater *F_u,SCB_* and larger *δ* at the maximum displacement, and yet both the *F_slip_* and *D*_1_ show opposite trends. Moreover, it should be noted that the maximum self-centering capacity ratio is 89.38%, showing that the self-centering SMA brace has an excellent re-centering capacity.

## 4. Numerical Results

The SIMULINK toolbox from MATLAB was used to simulate the seismic performance of the self-centering SMA brace, and the numerical and test results will be compared with the same conditions.

### 4.1. Numerical Model of Self-Centering SMA Brace

In Equation (3), the force of the SMA wires can be expressed by:

(5) $$F_{SMA}=\sigma_{SMA}A_{s}$$

where *σ_SMA_* and *A_s_* are the stress and cross-sectional area of the SMA wires, respectively.

According to the improved Graesser and Cozarelli model by Graesser [24] and Qin [21], the *σ_SMA_* can be calculated as follows:

(6) $$\dot{\sigma }=E\left[\dot{\varepsilon }-\left|\dot{\varepsilon }\right|{\left(\left.\frac{\sigma -\beta }{Y}\right)\right.}^{n-1}\left(\left.\frac{\sigma -\beta }{Y}\right)\right.\right]$$

(7) $$\beta =E\alpha \left\{\varepsilon_{in}-\frac{\sigma }{E}+f_{T}{\left|\varepsilon \right|}^{c}erf(a\varepsilon )[u(-\varepsilon \dot{\varepsilon })]+f_{M}{\left[\varepsilon -\varepsilon_{M_{f}}\mathrm{sgn}(\varepsilon )\right]}^{m}[u(\varepsilon \dot{\varepsilon })][u(\left|\varepsilon \right|-\varepsilon_{M_{f}})]\right\}$$

where *ε*, *E*, and *Y* are the strain, elastic modulus, and yield stress of the SMA wire, respectively; *n* is a constant controlling the sharpness of the transition from the elastic state to the phase transformation; *β* is the one-dimensional back stress; *α* is equal to *E_y_*/(*E*−*E_y_*); *E_y_* is the slope of stress–strain curve in the plastic range; *ε_in_* is the inelastic strain; *f_T_*, *a,* and *c* are the material constant controlling the type and size of the hysteresis, the amount of elastic recovery, and the slope of the unloading stress plateau, respectively; *ε_Mf_* is the Martensite finish transformation strain; and *f_M_* and *m* are the constants controlling the Martensite hardening curve.

The error function *erf* (x), Heaviside function *u* (x), and sigh function sign (*x*) can be expressed as [21]:

(8) $$erf(x)=\frac{2}{\sqrt{x}}\int_{0}^{x}e^{-t^{2}}dt$$

(9) $$u(x)=\left\{\begin{array}{ll} 1 & \left(x\geq 0\right)\\ 0 & \left(x<0\right) \end{array}\right.$$

(10) $$\mathrm{sgn}(x)=\left\{\begin{array}{ll} +1 & x>0\\ 0 & x=0\\ -1 & x<0 \end{array}\right.$$

Therefore, the numerical model of the SMA wires will be revealed in detail using Equations (7) and (8) during the numerical analysis.

To accurately simulate the mechanical properties of the slip component, the Bouc-Wen model [25] is presented and described by:

(11) $$F_{slip}=\lambda kd+\left(1-\lambda \right)kD_{y}Z$$

where *d*, *k*, *D_y_*, *λ,* and *Z* are the deflection, initial stiffness, yield displacement, ratio of plastic and elastic stiffness, and non-dimensional displacement, respectively.

The first-order non-dimensional displacement equation yields can be expressed by:

(12) $$\dot{Z}D_{y}=-\gamma \left|\dot{d}\right|Z{\left|Z\right|}^{\eta -1}-\beta \dot{d}{\left|Z\right|}^{\eta }+\theta \dot{d}$$

where *γ*, *β*, and *θ* are the parameters to control the shape and size of the hysteresis curve, and *η* is a scalar value to govern the smoothness of the transition from the elastic stage to the plastic stage.

In addition, according to the material properties of the SMA wires in Section 2.2.1 and the test results in Section 3, the model parameters of the SMA wire and slip model can be determined and are listed in Table 6.

### 4.2. Comparison of Test and Numerical Results

#### 4.2.1. Loading Rate

**Figure 14:**
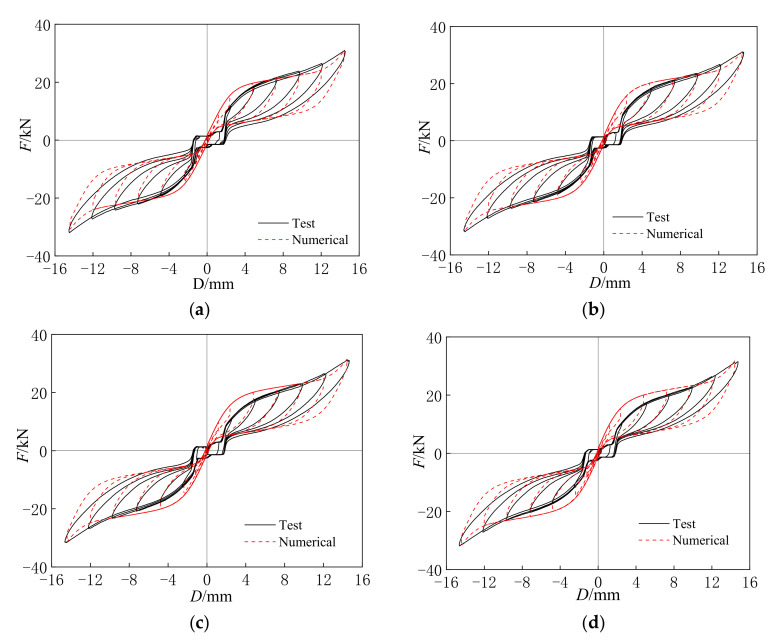
(**a**) SCB-12-0; (**b**) SCB-18-0; (**c**) SCB-24-0; (**d**) SCB-36-0. Comparison between test and numerical hysteresis curves under different loading rates.

The hysteresis curves of the numerical results were simulated using the improved Graesser model and Bouc–Wen model, which need to combine with the main parameters in Table 2 and Table 6. Figure 14 displays the comparison of the test and numerical results under different loading rates. At the applied displacements of 2.40 mm and 4.80 mm for specimens SCB-12-0, SCB-18-0, SCB-24-0, and SCB-36-0, a great difference in the hysteresis curves between the test and numerical results can be observed, which can be explained by the existing errors between the slip bolt and slip hole in the test model. However, both the test and numerical hysteresis curves have almost the same initial stiffness for all of the specimens at the different loading rates. As the applied displacement increases, the hysteresis curves between the test and numerical results for each specimen are very close, and only a small error exists near $\sigma_{s}^{MA}$, which is the same as the error for the SMA wire at the different loading rates [22].

For the different loading rates, the secant stiffness and energy dissipation coefficients of the specimens obtained from the test and numerical results with the applied displacement values ranging from 2.40 mm to 14.40 mm are shown in Table 7 and Table 8. At the displacements of 2.40 mm and 4.80 mm, the maximum error between the test and numerical secant stiffness is 31.76%. Meanwhile, the maximum error is also 56.63% for the energy dissipation coefficient. The main reason for the error may be due to the difference between the slip bolt and hole in the test brace. As the displacement increases from 7.20 mm to 14.40 mm, the maximum errors in the secant stiffness and energy dissipation coefficient between the test and numerical results are 5.68% and 8.02%, respectively, showing good accuracy. Based on the above analysis, the presented numerical model determined from Equations (3)–(12) can be used to simulate the seismic performance of self-centering SMA braces with different loading rates.

#### 4.2.2. Initial Strain

**Figure 15:**
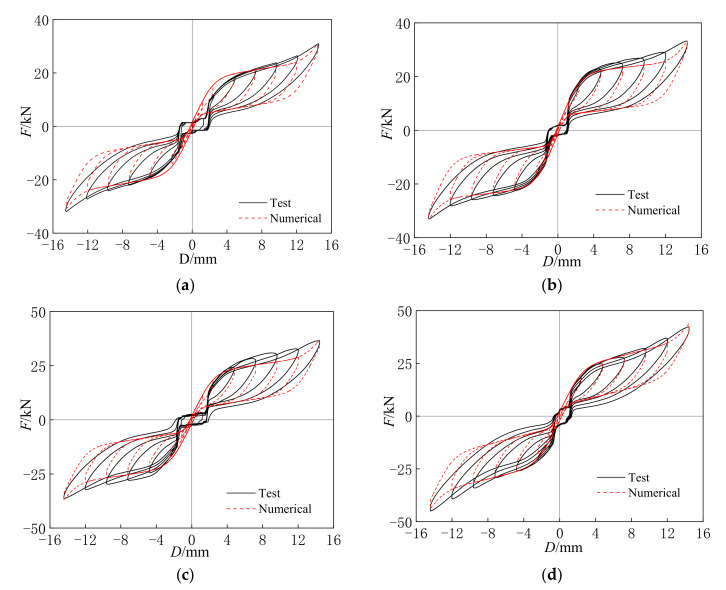
(**a**) SCB-12-0; (**b**) SCB-12-25; (**c**) SCB-12-50; (**d**) SCB-12-100. Comparison between test and numerical hysteresis curves under different initial strains.

Figure 15 shows the comparison between the test and numerical results under different initial strains at the applied displacements ranging from 2.40 mm to 14.40 mm. At the displacements of 2.40 mm and 4.80 mm for specimens SCB-12-0, SCB-12-25, SCB-12-50, and SCB-12-100, an obvious contrast between the test and numerical results can be observed, and the cause of the contrast is the same as it is with the loading rates. As the displacement increased from 7.20 mm to 14.40 mm, the hysteresis curves of the specimens obtained by the test results all show close agreement with the numerical results.

The secant stiffness and energy dissipation coefficient of the specimens calculated from test and numerical results are shown for the different initial strains in Table 9 and Table 10. At the displacement of 2.40 mm, the maximum errors of the secant stiffness and energy dissipation coefficient between the test and numerical results are 54.95% and 31.76%, respectively. As the strain amplitude increases, the numerical results of the secant stiffness and energy dissipation coefficient for all of the specimens become gradually closer to the test results, which have a maximum error of 7.64%, showing great agreement. Therefore, the proposed numerical model can also be used to analyze the seismic performance of the self-centering SMA braces with different initial strains.

## 5. Conclusions

In this paper, the cyclic loading test and numerical analysis were studied to carry out an innovative self-centering brace with the effect of the loading rate and initial strain, and the seismic performance of the brace was investigated. The following conclusions can be obtained:

(1) The proposed self-centering SMA brace has an excellent energy dissipation capacity, ductility, and self-centering capacity. The hysteresis curve can be idealized as a flag-shape with small residual deformation, and the self-centering capacity ratio reached 89.38%.
(2) Loading rate: As the loading rate increased, the ultimate axial force and secant stiffness coefficient increased gradually, the residual deformation decreased slightly, and the maximum *E_i_*, and the equivalent damping coefficient at each test case was able to be observed at the loading rate of 0.0018 s^−1^.
(3) Initial strain: The ultimate axial force, secant stiffness coefficient, and energy dissipation coefficient indeed increased gradually as the initial strain increased from 0 to 0.01, but the maximum equivalent damping coefficient of the self-centering SMA brace appears at the initial strain of 0.
(4) The improved numerical model combined with the Graesser model and Bouc–Wen model can be used to analyze the seismic performance of self-centering SMA braces with different loading rates and initial strains, and the numerical results are consistent with the test results under the same conditions.

## Figures and Tables

**Table 1:** Main specimen parameters.

No.	Specimens	Torque Value/N·M	SMA Area/mm^2^	Loading Rate/s^−1^	Initial Strain/%
1	SCB-12-0	10	43.96	0.0012	0
2	SCB-18-0	10	43.96	0.0018	0
3	SCB-24-0	10	43.96	0.0024	0
4	SCB-36-0	10	43.96	0.0036	0
5	SCB-12-25	10	43.96	0.0012	0.25
6	SCB-12-50	10	43.96	0.0012	0.50
7	SCB-12-100	10	43.96	0.0012	1.00

**Table 2:** Main parameters of SMA wire with different influencing factors.

Influencing Factor	Value	$\bar{\varepsilon_{L}}$	$E_{A}/\mathrm{MPa}$	$\sigma_{s}^{AM}/\mathrm{MPa}$	$\sigma_{f}^{AM}/\mathrm{MPa}$	$\sigma_{s}^{MA}/\;\mathrm{MPa}$	$\sigma_{f}^{MA}/\mathrm{MPa}$
Loading rate/s^−1^	0.0012	0.06	47000	492.81	703.70	277.53	117.89
	0.0018	0.06	49000	493.94	712.23	288.85	117.49
	0.0024	0.06	50000	495.30	720.81	295.19	116.55
	0.0036	0.06	51000	496.30	730.62	303.79	116.24
Pre-tensioned/*Δε*	0.0025	0.06	50000	490.81	742.35	288.29	119.44
	0.0050	0.06	51000	484.68	776.27	285.48	135.37
	0.0075	0.06	52000	475.81	807.56	274.28	146.18
	0.0100	0.06	53000	433.86	813.31	258.74	156.65

**Table 3:** Mechanical properties of steel plates.

No.	Thickness/mm	Yield Strength/MPa	Tensile Strength/MPa	Young’s Modulus/GPa	Elongation/%
1	8	365	545	206	23.1
2	15	372	556	209	25.2

**Table 4:** Test cases of self-centering SMA brace specimens.

Test Case	Specimens	Loading Rate/s^−1^	Initial Strain/%	Loading Displacement/mm	Loading Cycles
1	SCB-12-0	0.0012	0	1.20, 2.40, 4.80, 7.20, 9.60, 12.00, 14.00	1
2	SCB-18-0	0.0018	0	1.20, 2.40, 4.80, 7.20, 9.60, 12.00, 14.00	1
3	SCB-24-0	0.0024	0	1.20, 2.40, 4.80, 7.20, 9.60, 12.00, 14.00	1
4	SCB-36-0	0.0036	0	1.20, 2.40, 4.80, 7.20, 9.60, 12.00, 14.00	1
5	SCB-12-25	0.0012	0.25	1.20, 2.40, 4.80, 7.20, 9.60, 12.00, 14.00	1
6	SCB-12-50	0.0012	0.50	1.20, 2.40, 4.80, 7.20, 9.60, 12.00, 14.00	1
7	SCB-12-100	0.0012	1.00	1.20, 2.40, 4.80, 7.20, 9.60, 12.00, 14.00	1

**Table 5:** Performance indices of self-centering SMA braces.

Specimen	*F_u,SCB_*/kN	*F_slip_*/kN	*F_SMA_*/kN	*D*/mm	*D*_1_/mm	*δ*/%
SCB-12-0	31.08	1.56	29.52	14.40	1.97	86.32
SCB-18-0	31.49	1.52	29.97	14.40	1.90	86.81
SCB-24-0	31.75	1.46	30.29	14.40	1.87	87.01
SCB-36-0	32.23	1.34	30.89	14.40	1.82	87.36
SCB-12-25	33.21	1.76	31.45	14.40	1.89	86.88
SCB-12-50	36.80	2.15	34.65	14.40	1.69	88.26
SCB-12-100	42.24	2.83	39.39	14.40	1.53	89.38

**Table 6:** Determined the parameters of the SMA wire and slip model.

SMA Wire		Slip Component
*L* = 240mm	*n* = 3	*λ* = 0.0001, *η* = 4
*A* = 43.96 mm^2^	*ε*_Mf_ = 0.04	*k* = 12000
*α* = 0.019	*f_m_* = 42500	*D_y_* = 0.1
*m* = 3	*m* = 3	*γ* = 0.5
*c* = 0.001	*a* = 240	*β* = 0.5
*f_t_* = 0.79		*θ* = 0.9

**Table 7:** Comparison of secant stiffness under different loading rates.

Strain Amplitude/%	Loading Rate											
	0.0012/s			0.0018/s			0.0024/s			0.0036/s		
	Tes.	Num.	Error/%	Tes.	Num.	Error/%	Tes.	Num.	Error/%	Tes.	Num.	Error/%
1	4.87	6.42	31.76	5.28	6.48	22.87	5.40	6.53	20.88	5.70	6.58	15.45
2	3.63	4.09	12.68	3.74	4.17	11.47	4.07	4.18	2.77	4.20	4.19	0.11
3	2.77	2.85	2.66	2.98	3.02	1.37	3.22	3.10	3.63	3.37	3.29	2.32
4	2.39	2.26	5.68	2.47	2.42	2.28	2.60	2.54	2.55	2.73	2.66	2.45
5	2.32	2.24	3.28	2.39	2.30	3.74	2.47	2.42	2.08	2.54	2.49	2.10
6	2.30	2.21	4.23	2.37	2.29	3.65	2.41	2.32	3.39	2.47	2.41	2.22

**Table 8:** Comparison of energy dissipation coefficient under different loading rates.

Strain Amplitude/%	Loading Rate											
	0.0012/s			0.0018/s			0.0024/s			0.0036/s		
	Tes.	Num.	Error/%	Tes.	Num.	Error/%	Tes.	Num.	Error/%	Tes.	Num.	Error/%
1	8.76	3.94	54.95	9.11	3.98	56.35	9.19	3.99	56.55	9.27	4.02	56.63
2	37.00	42.03	13.59	37.54	43.00	14.56	38.07	43.25	13.62	38.14	43.50	14.05
3	88.33	95.08	7.64	91.83	99.19	8.02	93.43	100.78	7.87	95.31	102.77	7.83
4	164.75	173.86	5.53	167.97	178.94	6.53	170.15	179.28	5.36	172.06	182.42	6.02
5	246.25	257.97	4.76	247.37	259.30	4.82	248.67	261.68	5.23	251.25	266.05	5.89
6	317.37	327.92	3.32	320.81	334.75	4.35	310.73	319.38	2.79	291.13	296.80	1.95

Note. Error = (Numerical − Test)/Test: ‘Tes.’ and ‘Num’ denote the test and numerical results, respectively.

**Table 9:** Comparison of energy dissipation capacity under different initial strains.

Strain Amplitude/%	Initial Strain/%											
	0			0.25			0.5			1		
	Tes.	Num.	Error/%	Tes.	Num.	Error/%	Tes.	Num.	Error/%	Tes.	Num.	Error/%
1	8.76	3.94	54.95	14.25	9.17	35.65	17.96	10.47	41.71	21.29	13.77	35.33
2	37.00	42.03	13.59	56.49	60.06	6.325	62.61	66.37	6.00	69.20	73.22	5.80
3	88.33	95.08	7.64	117.98	124.16	5.23	124.84	130.97	4.91	143.83	150.35	4.53
4	164.75	173.86	5.53	186.16	194.60	4.54	198.96	207.10	4.09	212.85	220.99	3.82
5	246.25	257.97	4.76	256.61	266.45	3.83	267.08	276.25	3.43	275.00	283.34	3.03
6	317.37	327.92	3.32	318.48	328.74	3.22	341.65	350.30	2.53	342.98	351.32	2.43

**Table 10:** Comparison of secant stiffness under different initial strains.

Strain Amplitude/%	Initial Strain/%											
	0			0.25			0.5			1		
	Tes.	Num.	Error/%	Tes.	Num.	Error/%	Tes.	Num.	Error/%	Tes.	Num.	Error/%
1	4.87	6.42	31.76	7.66	7.31	4.49	7.98	7.69	3.70	8.70	8.28	4.78
2	3.63	4.09	12.68	5.12	4.90	4.36	5.48	5.11	6.83	5.76	5.42	5.83
3	2.77	2.85	2.66	3.73	3.63	2.69	3.94	3.78	3.93	4.26	4.11	3.49
4	2.39	2.26	5.68	2.98	2.85	4.16	3.43	3.28	4.44	3.62	3.47	4.10
5	2.32	2.24	3.28	2.56	2.45	4.11	2.89	2.75	4.71	3.30	3.17	3.90
6	2.30	2.21	4.23	2.42	2.35	3.09	2.66	2.58	3.18	3.11	3.05	2.13

## Data Availability

The data used to support the findings of this study are available from the corresponding author upon request.
